# Alternative to animal experimentation in pharmacology teaching: Development and validation of an equivalent digital learning tool

**DOI:** 10.1002/prp2.908

**Published:** 2022-02-11

**Authors:** Roland Lawson, Sophie Leymarie, Claire Nikitopoulos, Antoine Humeau, Hichem Bouchenaki, Jean‐Luc Duroux, Laurent Fourcade, Sandrine Karam, Nicolas Picard, Claire Demiot

**Affiliations:** ^1^ Faculté de Pharmacie Département de pharmacologie Univ. Limoges Inserm U1248 Limoges France; ^2^ CHU Limoges Pôle Thorax Abdomen Limoges France; ^3^ Faculté de Médecine Département Universitaire d’Enseignement Numérique en Santé (DUENES) Univ. Limoges Limoges France; ^4^ Faculté de Pharmacie Département de pharmacologie Univ. Limoges EA 6309 Limoges France; ^5^ Faculté de Pharmacie Département de biophysique et mathématiques Univ. Limoges EA 1069 Limoges France; ^6^ Medialab (Pôle Formation et Vie Etudiante) Univ. Limoges Limoges France

**Keywords:** animal experimentation, digital teaching tool, knowledge acquisition, motivation, preclinical pharmacology teaching

## Abstract

Regarding animal experiments in pharmacology teaching, ethical considerations led us to examine an alternative approach to the use of living animals. This study aimed to assess whether digital tools could replace live animal experiments in terms of motivation and knowledge acquisition. The study was carried out with students enrolled in the 5th year of the industry/research stream at the Faculty of Pharmacy of the University of Limoges. The participants were randomly assigned to groups of traditional or digital teaching methods, with the common theme of the class being the effect of a diuretic agent (furosemide) in rats. The scenario and learning objectives were identical for the two groups. Before the class and after randomization, the acceptance of the digital educational material was assessed with a scale, which predicts the acceptability of users according to individual dimensions and social representations, followed by the assessment of the motivation by a situational motivation scale (SIMS) for both groups. After the class, the students’ motivation was assessed by a questionnaire based on Deci and Ryan's self‐determination theory. In the end, the participants were evaluated for homogeneity, based on general knowledge of renal pharmacology, and for knowledge acquisition concerning specific knowledge related to this teaching session. This study revealed a good acceptance of the digital tool and a good motivation toward the digital method among all the students. It found the two teaching methods (digital and traditional) to be equivalent in terms of motivation and knowledge acquisition. In our study, digital pedagogical tools as an alternative to live animals did not affect students’ motivation and knowledge acquisition.

## Graphical abstract

This study reveals a good acceptance of the digital tool and good motivation towards digital method in all of the students. It found equivalent the two teaching methods, digital and traditional in terms of motivation and knowledge acquisition.

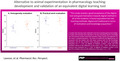

**Abbreviations**
PIE: planning‐implementation‐evaluation
SIMS: situational motivation scale
SMART: specific, measurable, assignable, realistic, and time‐related

## 1 INTRODUCTION

In recent times, animals have been recognized under French law (the French civil code) as “living sentient beings”.^1^ Given this, the Faculty of Law and Economics at the University of Limoges established a university degree course on the rights of animals in 2016. In the same perspective, the teaching department of pharmacology at the Faculty of Pharmacy undertook an ethical review on the necessity of animal use in the setting of undergraduate teaching. Therefore, taking into consideration the three R’s rule (i.e., reduction, refinement, replacement),^2^ parties involved in animal experimentation sought to reduce the number of animals in teaching notably by implementing digital tools.^3^ Digital tools allow a diversity of resources provided to students and the development of new interactive forms of delivery. In addition, numerous studies have identified digital tools as equivalent or possibly superior to traditional learning in medical training in terms of knowledge acquisitions, learning skills, developing attitudes, and assessment.^4^, ^5^, ^6^, ^7^

Undergraduate pharmacology teaching generally requires conception and understanding of scientific protocols used for studying the mechanisms of action of drugs. The teaching activity used for this study was a task on “the effect of furosemide ^8^ on diuresis in the rat.” Our team traditionally followed a “planning‐implementation‐evaluation” (PIE) approach^9^ associated to live animal experiments for in vivo practical pharmacology training. PIE is a dynamic process, which is crucial for accurate curriculum development. The initial “planning” step identifies supporting materials and addresses the needs that are a priority for the learners, the teachers, the community, and the society. The “implementation” step puts into practice the designed curriculum. The “evaluation” step follows to assess if the intended knowledge, skills, and attitudes are achieved. Therefore, our study aimed to evaluate whether the use of digital resources as educational tools following the same PIE approach could replace live animal experiments for this teaching session. More precisely, the main objective was to find out whether traditional and digital teaching methods had a different influence on students’ pre‐ and post‐instructional motivation and knowledge acquisition. To address this objective, a randomized study was conducted.

Before the teaching and after the randomization, we verified that the two groups were homogeneous regarding the acceptance of the digital tool, then an analysis of the situational motivation was carried out in order to know if the fact of knowing the “digital or traditional” group could influence the motivation to carry out the teaching. After the teaching, their motivation was evaluated with a questionnaire adapted to the animal experiment based on the self‐determination theory of Deci and Ryan.^10^ The performance (knowledge acquisition) was evaluated by a final exam after having verified that the two groups were homogeneous in terms of general level.

## 2 MATERIALS AND METHODS

### 2.1 Participants and study design

**FIGURE 1:**
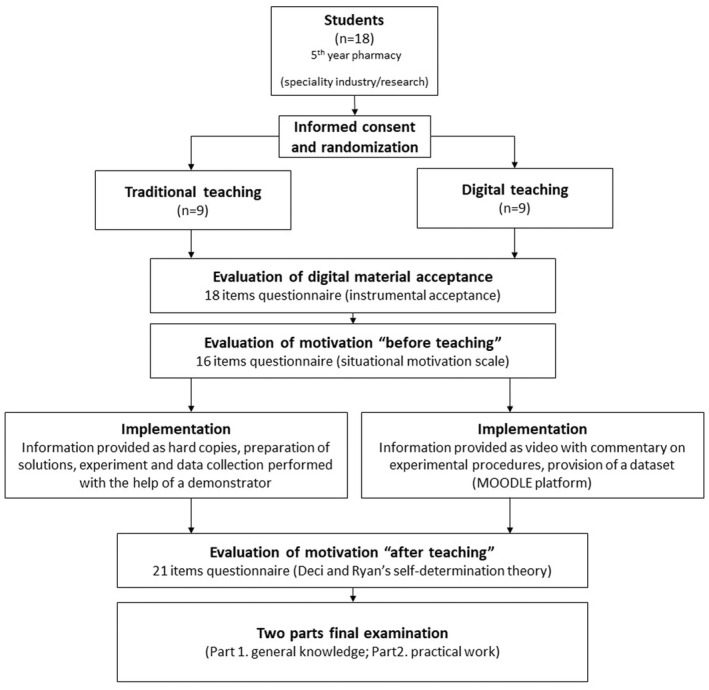
Flow chart of the study

The study was carried out with 18 students enrolled in the 5th year of the industry/research stream at the Faculty of Pharmacy of the University of Limoges in 2019–2020. They were randomly assigned to traditional or digital teaching methods with Excel random function. The two groups studied during a practical class, the effect of furosemide on diuresis in rats. The learning objectives, which outlines the knowledge, skills, and/or attitudes the learners will gain from the educational activity,^11^ were the same for both groups: (1) To know the steps involved in an experiment carried out in an experimental animal to answer a scientific question; (2) to know how to use experimental data and present it in the form of a graph; (3) to identify the different parameters or groups to compare to answer a scientific question; (4) to compare parameters or groups using appropriate statistical tests; (5) to calculate concentrations or volumes of solutions of diuretic agent to administer starting from powder form or dosage form; and (6) to acquire critical thinking and writing skills in order to discuss the results in relation to the published data. The only difference was the teaching method used: traditional or digital (Figure 1 and Table 1).

**TABLE 1:** Planning‐implementation‐evaluation approach for the practical class according to the traditional or digital methods

Title of the teaching activity	Study of the effect of furosemide on diuresis in the rat	
Teaching modalities	Traditional delivery	Digital delivery
Planning ‘think before and after’		
Existing knowledge	Renal physiology	
	Pharmacology and therapeutic effect of diuretic agents	
	Ethic of animal experimentation	
	Regulation of the study of new candidate drugs in animals	
	Statistical methods in animal experimentation	
	Literature searching and formatting of references	
Competencies to be acquired	Know and understand the concept of a scientific protocol used to study the effect of a drug in an experimental animal	
Objectives	1. Know the steps involved in an experiment carried out in an experimental animal to answer a scientific question	
	2. Know how to use experimental data and present it in the form of a graph	
	3. Identify the different parameters or groups to compare to answer a scientific question	
	4. Compare parameters or groups using appropriate statistical tests	
	5. Calculate concentrations or volumes of solutions of diuretic agent to administer starting from powder form or dosage form	
	6. Acquire critical thinking and writing skills in order to discuss the results in relation to published data	
Implementation ‘building competencies’		
Revise knowledge of renal physiology and the pharmacology of diuretics	Documents provided by the teaching staff in hard copy	Documents accessible online via a digital platform dedicated to this course (MOODLE)
	10 practice MCQs given as hard copy to be answered individually followed by correction and discussion	10 practice MCQs available online via MOODLE, allowing multiple attempts. This activity was self‐administered and validated when the student achieved 100% of correct responses, following the pedagogical principle of learning by error
Presentation of the learning objective	Oral presentation and information provided as hard copies	Video presentation via MOODLE and provision of a video with commentary
Presentation of the objective of the experiment		
Presentation of the materials and methods		
Calculation of volumes and concentrations of solutions to be administered and understanding preparation procedures	Performed on data obtained from oral and hard copy information provided, then recorded and corrected	Performed on data obtained from the online video and slideshow then recorded and corrected
Preparation of solutions	Done by the students	Not done (not applicable)
Carrying out the experiment and producing dataset	Performing the experiment with the help of a demonstrator who is the only person, in this experiment, permitted to perform procedures with the experimental animals	Presentation of the experimental procedure via a videographic document and provision of a data file (via MOODLE) with the results (cumulative 24 h urine volumes) and instructions on the preparation of the dataset to be downloaded
	Collected data for cumulative 24 h urine volumes presented as a dataset	
Analysis of results	Presenting the data to be analyzed as a graph	
	Using the R Commander (Rcmdr) software program to perform statistical analysis of the data	
Writing the discussion of the results including appropriate bibliographic references	Identical access to references for writing the discussion with the aid of instructions from the lecturer/demonstrator	
Student evaluation	Analysis of results from an incomplete scientific paper and proposal of an experimental protocol and some elements of the methodology	

### 2.2 Planning‐implementation‐evaluation: The digital versus traditional teaching method

The design and construction of the practical class were performed according to the PIE approach. Planning for the two teaching methods was synchronized and the objectives were equivalent in the two groups. Learning objectives were formulated according to the specific, measurable, assignable, realistic, and time‐related (SMART) pedagogical method.^12^ Differences in the implementation of the two teaching methods are outlined below. Supporting material consisting of paper and oral instructions were provided for the traditional group. This group prepared the injection solution in a face‐to‐face class, then observed the experiment that was carried out by the demonstrator, including data collection from the animals in metabolic cages (equipped with a graduated reservoir for urine collection). The digital group received face‐to‐face teaching in a computer laboratory and followed the experiment using digital support material, including animated pictures, interactive slides, and chronological videos of a demonstrator performing the experiment with Moodle (version 2.9) platform. They carried out calculations including the mass of the drug to be weighed to prepare the injection solution per the number of animals to be used in the experiment, but without carrying out the actual weighing or solution preparation. They obtained the raw data (urinary volumes at 1, 3, 6, and 12 h after administration of solutions) via a provided data file. The students submitted reports (graphs and discussions) on paper for the traditional group and on the Moodle platform for the digital group. For both groups, a two‐part final examination was performed after the class. The first part was related to the homogeneity for the general knowledge of renal pharmacology and the second part on the specific knowledge taught during this session and was based on a scientific paper that allowed the evaluation of the six learning objectives in the two groups (Figure 1 and Table 1).

### 2.3 Evaluation of acceptance of a digital learning tool

The students responded to a questionnaire to assess digital educational material acceptance^13^ after receiving their consent and being randomized. This questionnaire is based on a scale, which predicts the acceptability of users according to individual dimensions and social representations. It has 18 items that evaluated 6 parameters: overall value, usefulness for the student, usability, injunction, usefulness for the teacher for training, and value for the student. Each item was scored on a 7‐point scale from 1 “completely disagree” to 7 “completely agree” (File S1). Data are represented by box‐whisker plots showing the median score and interquartile intervals for the different parameters. Statistical significance (*p* < .05) in the difference between the two groups was analyzed with the Wilcoxon test.

### 2.4 Evaluation of motivation

After the assignment to their groups, all students responded before (File S2) and after (File S3) the learning activity to a questionnaire on motivation. The before and after activity questionnaire were respectively composed of 16 and 21 items also scored on a 7‐point scale.

The before questionnaire, which aimed at evaluating the motivation of the students after being randomized, was based on the situational motivation scale (SIMS),^14^ which measures individual motivational orientation toward a particular activity. This multidimensional scale evaluates situational intrinsic motivation, extrinsic motivation (identified regulation, external regulation), and amotivation.^14^

The after activity motivation questionnaire was based on Deci and Ryan's theory of self‐determination (2002) and was adapted for the subject of animal experimentation in pharmacology.^10^ Self‐determination theory in an empirically derived theory of human motivation and personality in social contexts that differentiates motivation in terms of being autonomous and controlled. This questionnaire evaluated intrinsic motivation (intrinsic regulation), extrinsic motivation (integrated, identified, introjected, and external regulations), and amotivation. Data are represented by box‐whisker plots showing the median and interquartile interval for the different parameters.

### 2.5 Evaluation of knowledge acquisition

Evaluation of knowledge acquisition (see learning objectives, Table 1) was performed by a final examination, 1 month after the class. This final examination was a simultaneous two‐part evaluation. The first part aimed to assess with general questions on renal pharmacology, the homogeneity of the general knowledge level of the students, to ensure that the students have an equivalent level, and to avoid bias or misleading conclusions regarding the type of teaching method. The second part evaluated the specific knowledge that should be acquired through this activity (File S4). Each part of the final exam was marked out of 10 and mean scores ± standard deviation were calculated for both groups. The two groups were compared with the Mann–Whitney test for both general knowledge evaluation and final examination.

### 2.6 Nomenclature of targets and ligands

Key protein targets and ligands in this article are hyperlinked to corresponding entries in http://www.guidetopharmacology.org, the common portal for data from the IUPHAR/BPS Guide to PHARMACOLOGY,^15^ and are permanently archived in the Concise Guide to PHARMACOLOGY 2021/22.^16^

## 3 RESULTS

### 3.1 Attitude to the digital tools

**FIGURE 2:**
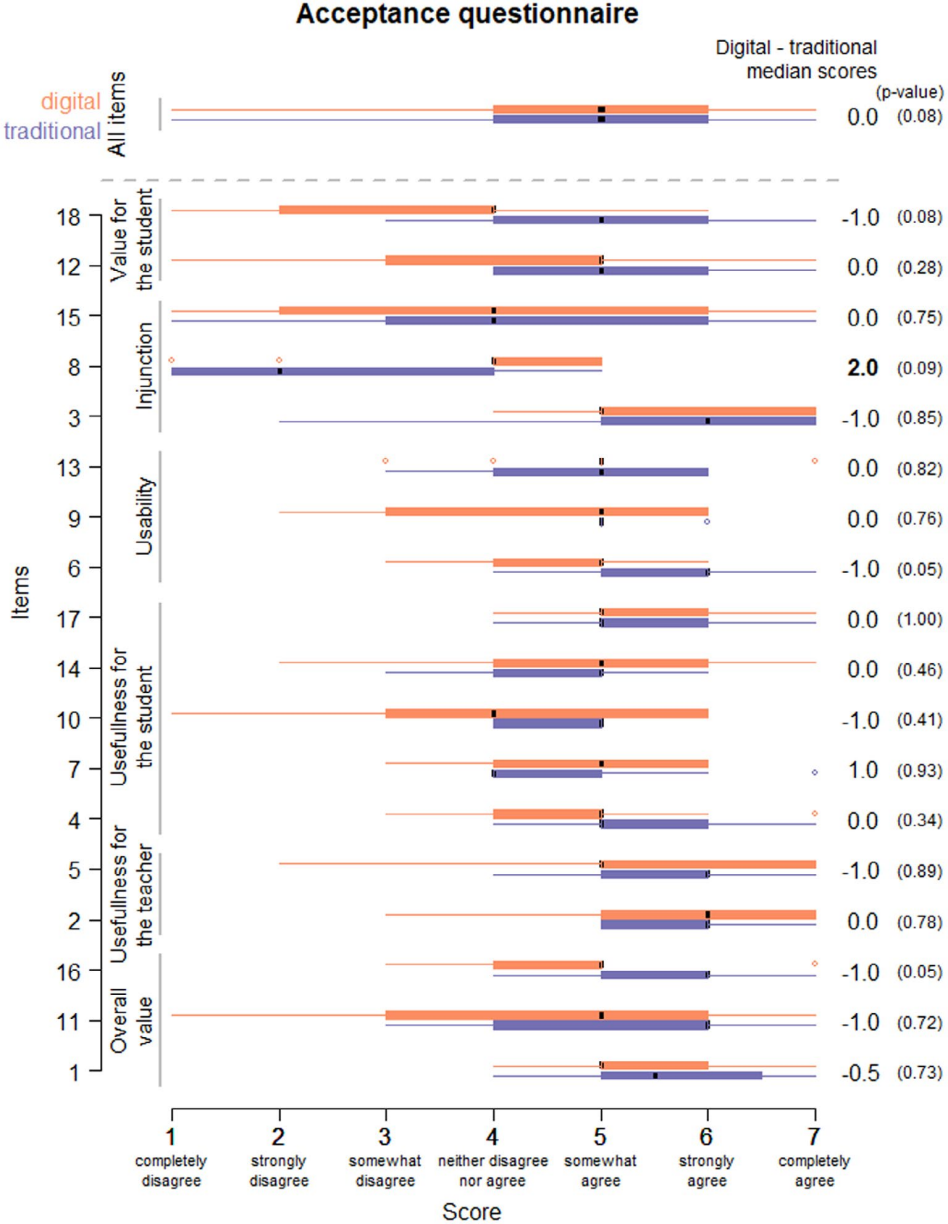
Digital material acceptance questionnaire (technology acceptance model questionnaire^13^) before the teaching. Boxplot of scores for each item (scaled from 1 to 7) in digital (orange) and traditional (purple) groups. The two upper boxplots merge all items per group. The difference between median scores of digital and traditional groups is at the right. The *p*‐value of the Wilcoxon test for the difference between the two groups is in parenthesis

Assessment of the participants’ understanding of digital tools showed that the two groups were equivalent in their relationship to digital tools. The median scores obtained for the evaluation questionnaire in both digital and traditional groups was 5 (min = 1, max = 7). Only item 8 ‘I use digital support materials because I hope it may be well‐regarded by my university’ had a median score that was two points higher on the evaluation scale in the digital group compared to the traditional group (Figure 2).

### 3.2 Evaluation of situational motivation before the activity

**FIGURE 3:**
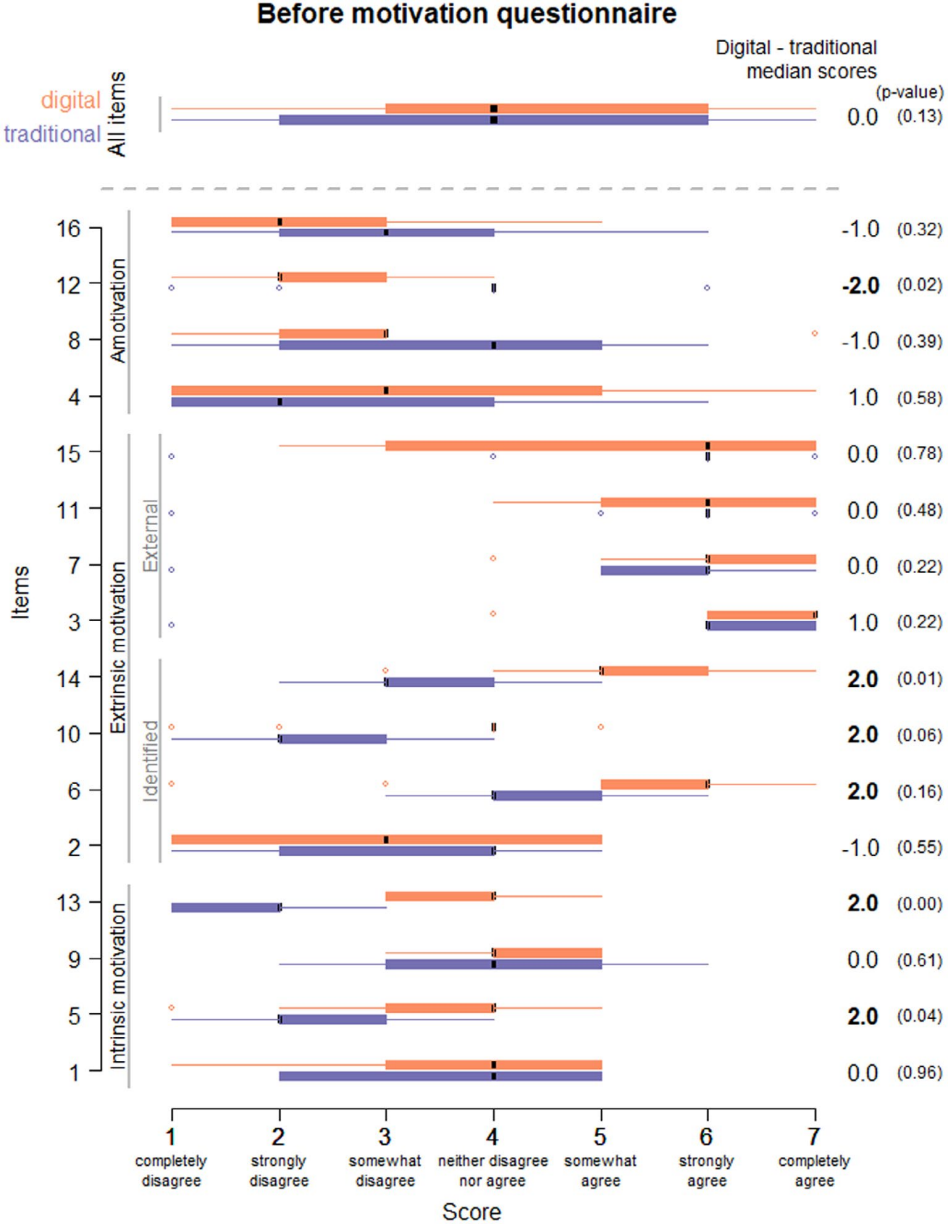
Situational motivation questionnaire before the teaching (SIMS questionnaire^14^). Boxplot of scores for each item (scaled from 1 to 7) in digital (orange) and traditional (purple) groups. The two upper boxplots merge all items per group. The difference between median scores of digital and traditional groups is at the right. The *p*‐value of the Wilcoxon test for the difference between the two groups is in parenthesis

The level of general situational motivation was similar in the two groups before the teaching activity. The median scores obtained for the evaluation questionnaire in both digital and traditional groups were 4 (min = 1, max = 7). The median scores in the digital group were higher by 2 points (on the evaluation scale) for items representing intrinsic motivation, specifically item 5: ‘I am happy doing this activity’ and item 13: “Because I find this activity enjoyable;” for items representing identified regulation of extrinsic motivation, item 6: “Because I feel this activity is important for me,” item 10: “I want to do this activity” and item 14: “I think this activity is good for me.” In opposite, a median score that was 2 points higher was found for the traditional group for amotivation, item 12: “I have to do this activity but I wonder why I have to do it” (Figure 3).

### 3.3 Evaluation of motivation after the activity

**FIGURE 4:**
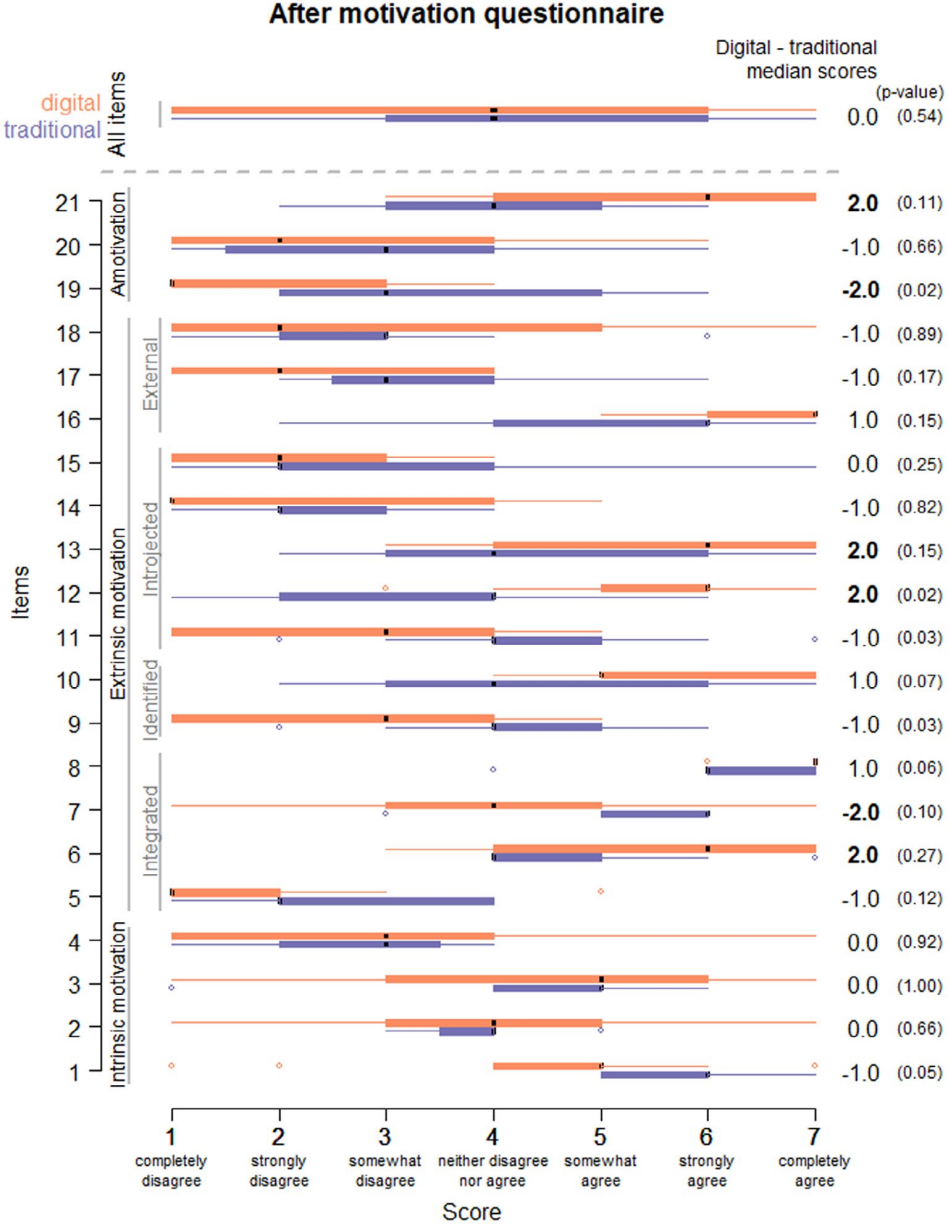
Motivation questionnaire after teaching based on Deci and Ryan's theory of self‐determination adapted for animal experimentation in pharmacology.^10^ Boxplot of scores for each item (scaled from 1 to 7) in digital (orange) and traditional (purple) groups. The difference between median scores of digital and traditional groups is at the right. The *p*‐value of the Wilcoxon test for the difference between the two groups in parenthesis

Median scores at this motivation questionnaire were 4 (min = 1, max = 7) for both the traditional group and the digital group. Thus, no effect was observed on the overall motivation of use of the new digital learning method for this activity. On the one hand, the median scores in the digital group were higher by 2 points for item 6, which evaluates integrated regulation of extrinsic motivation: “I think it is important to no longer work on animals in practical classes;” for, respectively, items 12 and 13, which evaluate introjected regulation of extrinsic motivation: “I prefer to use digital resources for practical classes as, according to my friends, it is better for animals” and “I have to do practical classes on animals though I would prefer not to,” and for item 21, which corresponds to amotivation: “It is demotivating to do experiments on animals.” Diversely, a median score that was 2 points higher was found for the traditional group: for item 7, which evaluates integrated regulation of extrinsic motivation: “I like the way in which digital resources have changed my professional life” and for item 19 which represents amotivation: “I don't see why we have to do practical classes using animals” (Figure 4).

### 3.4 Evaluation of knowledge acquisition

**FIGURE 5:**
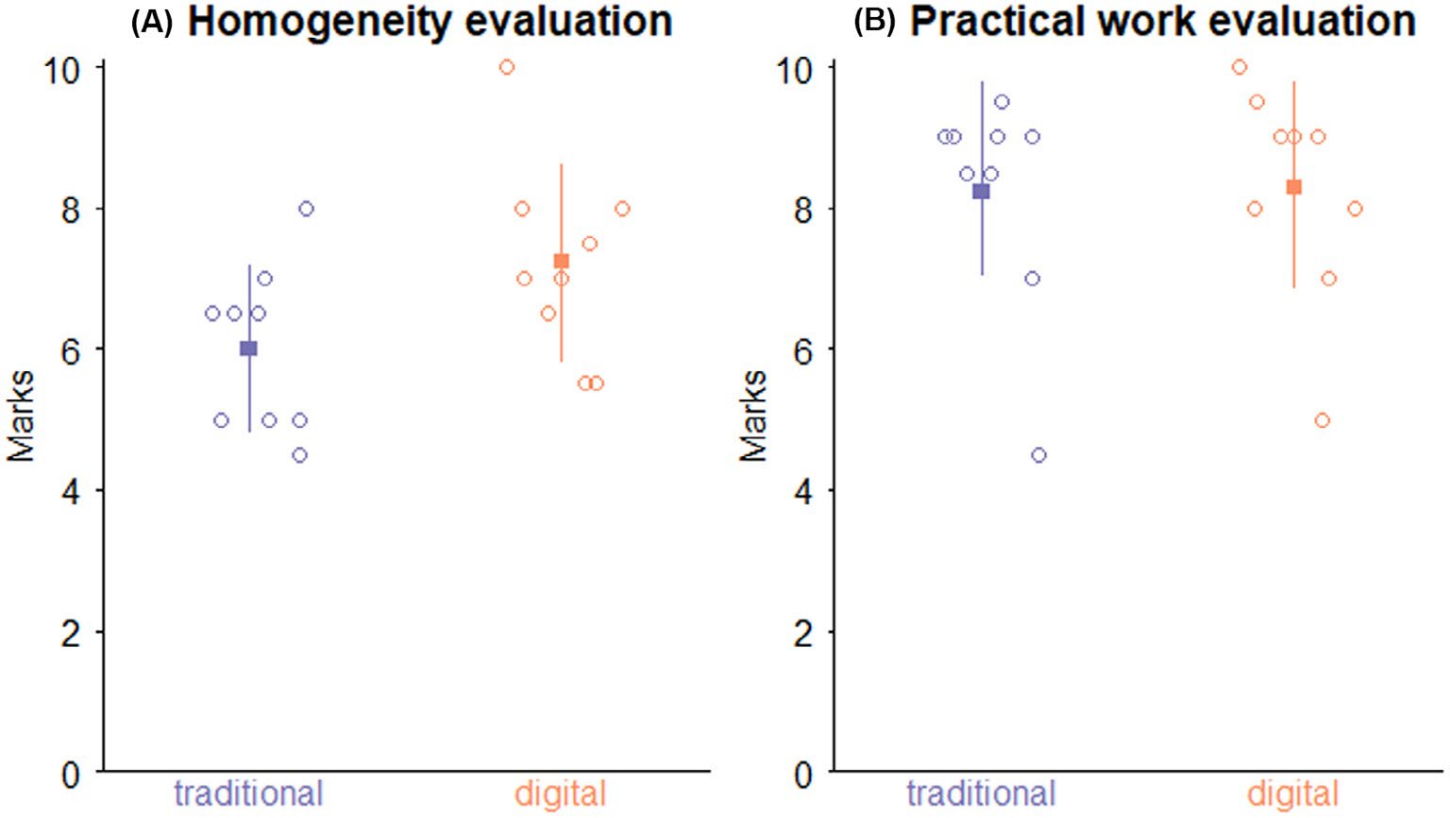
Evaluation of general knowledge and knowledge acquisition. (A) Scatter plot showing marks obtained in the test evaluating general knowledge; (B) Scatter plot showing marks obtained in the test evaluating specific knowledge from the practical session at the final evaluation. Black squares and segments are the means ± standard deviations. The means of traditional and digital groups are compared with the Student test with equal variance both for the knowledge evaluation and for the practical work evaluation. The results for knowledge evaluation are *t* = −2.01, 16 degrees of freedom, *p*‐value = .061 and 95% confident interval of the difference of the mean [−2.51 to 0.07]. The results for practical work evaluation are *t* = −0.08, 16 degrees of freedom, *p*‐value = .94, and 95% confident interval of the difference of the mean [−1.6 to 1.49]

Evaluation of the homogeneity of the two groups, in terms of the general knowledge of renal pharmacology, showed that the mean scores for the two groups of students were similar, the traditional group being 6.00 ± 1.2 compared to 7.2 ± 1.4 in the digital group (*p* > .05) (Figure 5A). A similar result was also observed for the final examination. Mean marks in the traditional group evaluation were 8.2 ± 1.6 versus 8.3 ± 1.5 for the digital group (*p* > .05) (Figure 5B).

## 4 DISCUSSION

The results obtained with the questionnaire on instrumental acceptability applied to the information technology used in education gave median scores of at least 5 for the digital group on 14 of the 18 items and on 15 of the 18 items for the traditional group. According to the Caron and Heutte model,^13^ this observation validates the fact that regardless of the assigned teaching method and the level of prior experience with digital teaching tools, the students had a similar overall understanding of digital tools as teaching methods and have similar acceptance of their use. This corroborates the idea that digital technologies are beneficial for teaching.^17^ Specifically, the items related to overall use, usefulness for the students themselves or for the teacher, value for the student, and usability are similarly scored for both groups. This suggests a perceived value of the use of digital materials during their training with the goal of personal accomplishment. This perceived ease of use of digital tools, suggests a certain desire of the students for digital materials and may be due to the speed and ease with which, students adapt to these tools. Students in both groups do not have resistance or psychological blocks to using digital materials during their training. As the study involved students in the 5th year of their degree whose prior experience was with traditional teaching methods, they were perhaps more inclined to experience a novel teaching modality. Indeed, the low score relating to the injunction dimension on item 8 reinforces these ideas (influence and social desirability).^18^ These relate to extrinsic motivation, that is, perceptions that influence a student to accept the digital support materials for reasons related to the era, to society, or an environment, rather than from internal convictions. In fact, it appears that students were influenced toward the use of digital tools by intrinsic arguments.

In terms of general situational motivation, which was evaluated using the SIMS (File S2) prior to the teaching activity, the median score of 4 in both groups confirmed a good level of motivation in students regardless of the teaching modality. Specifically, we found higher scores on the items related to intrinsic motivation and integrated (finding sources of self‐motivation) and introjected regulation (avoiding unpleasant consequences by feeling guilty) in the digital group. These results suggest that the prospect of participating in the class using digital teaching methods produced motivation more through integrated and introjected regulation in the digital group. Conversely, the traditional group had lower scores for the same parameters, these students being less motivated to take part in the activity via the traditional teaching modality.

In terms of amotivation, the difference in scores was greatest between the two groups, suggesting a trend toward an absence of motivation being more marked in the traditional group. This could be evidence of a relative lack of interest in traditional teaching activities with experimental animals, particularly as students are aware of the possibility of using other forms of teaching, notably digital methods.

Concerning motivation after the learning activity, even though the questionnaire that was used was different from that used prior to the class, it measured the same dimensions (intrinsic and extrinsic motivation), and the relatively high median score of 4 in both groups confirmed a good level of motivation for the teaching activity. Similarly, a comparison of scores for the various items confirmed that the motivation was mainly intrinsic. These results can be explained by the 5th year students’ experience of both traditional and digital teaching methods with well‐retained motivation. In addition, participation in the digital practical class seemed to reinforce the positive motivation towards the digital tool in the digital group. Items 6, 7, 12, and 13 (extrinsic motivation related to lack of interest in animal experimentation), where scores were higher in the digital group, raise the point that the ethical arguments in favor of less animal use in teaching may be predominant in these students. Moreover, this was confirmed by the higher score obtained for amotivation with item 21: “It is demotivating to carry out experiments on animals.” Conversely, in the traditional group, though the median score for item 19: “I do not see why it is necessary to do practical classes without animals,” was 3 (“somewhat disagree”) compared to a median score of 1 (“completely disagree”) in the digital group, the difference could be interpreted as in the favor of the pedagogical value of traditional modalities using animals, due to them having just completed this activity. This isolated result does not, however, call into question the students’ general motivation to use digital modalities. Although Michaut and Roche highlighted the contrasting and mixed results of the use of digital technology on the students’ performance during exams,^19^ the homogeneity of results obtained with respect to performance in the knowledge evaluation showed that the two teaching methods had comparable results, both allowing the same acquisition and retention of knowledge during the teaching activities. As stipulated by Meirieu,^20^ this result contrasts with the idea that “manual practice” leads to better retention of competencies, in this case, “learn and understand the concept of a scientific protocol in the setting of a study of the effect of a drug in animal experimentation.” In this context, watching a person in the situation of an animal experiment via a digital tool appears to stimulate cognitive processes in the students to the same extent as the traditional teaching method.

Regarding results on motivation, although it is not shown that digital tools have a positive impact on learning strategies,^19^ the situation of learning via a digital tool removed any ethical dilemma related to animal experimentation. In addition, the knowledge evaluation being performed one month after the learning activity reinforces the idea that there was retention and appropriation of knowledge acquired in the activity regardless of the teaching modality. It is worth mentioning that the technical skills related to handling rodents are not obligatory competencies for these students, but can be acquired through more specific training if so desired or needed.

## 5 CONCLUSION

This study reveals a good acceptance of the digital tool and good motivation toward the digital method in all of the students. It found equivalent the two teaching methods, digital and traditional in terms of motivation and knowledge acquisition. The reliability of the results seems based on the intrinsic aspects of the motivation and the ethical beliefs of the students, justifying their willingness to use digital tools in their learning and their university course. These results support the use of these tools, particularly in the teaching of practical classes, where hands‐on experimentation by students was traditionally the most developed. However, the success in the final examination and thus the retention of knowledge by students who took part in the digital teaching, does not tell us whether they would be able to carry out this type of experiment in the future, particularly in a professional situation, without having themselves done so during their training. Another limitation of this study is the relatively small sample size and data obtained from a single institution. In general, the difference in duration (approximatively 1 h) and learning environment (classic laboratory vs. computer laboratory) may explain some minor differences in student outcomes and attitudes. Further studies are required on these aspects to confirm both the ethical and pedagogical value of alternative digital tools to animal experimentation in university teaching.

## DISCLOSURE

The authors have no conflicts of interest to declare.

## AUTHORS’ CONTRIBUTIONS

R.L., S.L., C.N, and C.D. designed the study; R.L., C.N., and A.H. performed the analysis; R.L and CD drafted the manuscript. All authors aided in interpreting the results, worked on the manuscript, and approved the final manuscript.

## CONSENT STATEMENT

An oral presentation on the study and its objectives was delivered at the beginning to students. All participants gave their informed written consent for anonymous data to be collected, analyzed, and published as a part of this pedagogical research.

## ETHICS APPROVAL STATEMENT

The ethics committee of the University of Limoges (affiliated with the university hospital) approved this study with authorization number 341‐2019‐107. All animal care and experimental procedures during the traditional practical work session were performed in accordance with the guidelines for animal experimentation of the European Communities Council Directive (EU/63/2010) with the authorization number APAFIS#7843‐2016120109563578 v1.

## Supporting information

Supplementary Material

## Data Availability

The datasets used and/or analyzed during this study are available from the corresponding author on reasonable request.
