# Supplementary material for: Alternative to animal experimentation in pharmacology teaching: Development and validation of an equivalent digital learning tool
Source: Pharmacol Res Perspect. 2022 Feb 11;10(1):e00908. doi: 10.1002/prp2.908 (PMC8929359; doi:10.1002/prp2.908)
Supplement: Supplementary file 1 — Supplementary Material [file PRP2-10-e00908-s001.docx]

**Additional file 1: Scale of instrumental acceptance applied to information technology used in education, used by the students**

**French version used**

Lisez chaque affirmation avec attention. Puis, en utilisant l’échelle suivante, entourez le nombre qui décrit le mieux à quel point vous êtes en accord avec chaque affirmation.

**Utilisez l’échelle ci- dessous :**

| **Pas du tout d’accord**  **1** | **Très peu d’accord**  **2** | **Un peu d’accord**  **3** | **Moyennement d’accord**  **4** | **Assez d’accord**  **5** | **Fortement d’accord**  **6** | **Tout à fait d’accord**  **7** |
| --- | --- | --- | --- | --- | --- | --- |

**Etes-vous :**

- Une femme
- Un homme

**Avez-vous réalisé :**

- Le TP traditionnel
- Le TP numérique

| ***Affirmations sur l’utilité du support Numérique (SNum) en TP*** | | | | | | | |
| --- | --- | --- | --- | --- | --- | --- | --- |
| **1.** L’utilisation du SNum en TP me permet d’accomplir des tâches plus rapidement. | **1** | **2** | **3** | **4** | **5** | **6** | **7** |
| **2.** Je pense que le SNum en TP est utile pour former. | **1** | **2** | **3** | **4** | **5** | **6** | **7** |
| **3.** J’utilise le SNum en TP parce qu’on me le demande. | **1** | **2** | **3** | **4** | **5** | **6** | **7** |
| **4.** Le SNum en TP améliore ma capacité à appliquer la formation reçue. | **1** | **2** | **3** | **4** | **5** | **6** | **7** |
| **5.** Avec le SNum, je pense que c’est plus facile pour former. | **1** | **2** | **3** | **4** | **5** | **6** | **7** |
| **6.** C’est facile d’utiliser le SNum pour faire ce que je veux faire. | **1** | **2** | **3** | **4** | **5** | **6** | **7** |
| **7.** Le SNum me permet d’apprendre plus rapidement. | **1** | **2** | **3** | **4** | **5** | **6** | **7** |
| **8.** J’utilise le SNum parce que j’espère de son usage une certaine reconnaissance au sein de mon établissement. | **1** | **2** | **3** | **4** | **5** | **6** | **7** |
| **9.** C’est facile d’apprendre à utiliser le SNum en TP. | **1** | **2** | **3** | **4** | **5** | **6** | **7** |
| **10.** Avec le SNum, c’est plus facile d’apprendre en TP. | **1** | **2** | **3** | **4** | **5** | **6** | **7** |
| **11.** Je trouve le SNum utile pour mon projet d’étude. | **1** | **2** | **3** | **4** | **5** | **6** | **7** |
| **12.** Le SNum me permet de mieux mettre en œuvre mon projet d’étude. | **1** | **2** | **3** | **4** | **5** | **6** | **7** |
| **13.** Je trouve le SNum facile à utiliser en TP. | **1** | **2** | **3** | **4** | **5** | **6** | **7** |
| **14.** Avec le SNum, j’ai plus de facilité pour accéder à la formation reçue. | **1** | **2** | **3** | **4** | **5** | **6** | **7** |
| **15.** J’utilise le SNum en TP parce que les autres apprenants l’utilisent. | **1** | **2** | **3** | **4** | **5** | **6** | **7** |
| **16.** L’utilisation du SNum en TP me rend plus efficace. | **1** | **2** | **3** | **4** | **5** | **6** | **7** |
| **17.** Le SNum en TP est utile pour mon apprentissage. | **1** | **2** | **3** | **4** | **5** | **6** | **7** |
| **18.** Avec le SNum, je comprends mieux mon projet d’étude. | **1** | **2** | **3** | **4** | **5** | **6** | **7** |

**English translation of the French version**

Read each statement carefully. Then, using the following scale, circle the number, which best describes the extent that you agree with each statement.

**Use the scale below:**

| **Completely disagree** | **Strongly disagree** | **Somewhat disagree** | **Neither agree nor disagree** | **Somewhat agree** | **Strongly agree** | **Completely**  **agree** |
| --- | --- | --- | --- | --- | --- | --- |
| **1** | **2** | **3** | **4** | **5** | **6** | **7** |

**Are you:**

- Female
- Male

**You completed:**

- Traditional practical class
- Digital practical class

| ***Statements on the usefulness of digital support materials (DSM) in practical classes*** | | | | | | | |
| --- | --- | --- | --- | --- | --- | --- | --- |
| **1.** The use of DSM in practicals allows me to do tasks more quickly | **1** | **2** | **3** | **4** | **5** | **6** | **7** |
| **2.** I think DSM in practicals is useful for learning | **1** | **2** | **3** | **4** | **5** | **6** | **7** |
| **3.** I use DSM in practicals because it is a requirement | **1** | **2** | **3** | **4** | **5** | **6** | **7** |
| **4.** DSM in practical classes helps me to apply the information that is provided | **1** | **2** | **3** | **4** | **5** | **6** | **7** |
| **5.** With DSM I find it is easier to learn | **1** | **2** | **3** | **4** | **5** | **6** | **7** |
| **6.** It is easy to use DSM to do what I want to do | **1** | **2** | **3** | **4** | **5** | **6** | **7** |
| **7.** DSM lets me learn more rapidly | **1** | **2** | **3** | **4** | **5** | **6** | **7** |
| **8.** I use DSM because I hope it may be well-regarded by my university | **1** | **2** | **3** | **4** | **5** | **6** | **7** |
| **9.** It is easy to learn how to use DSM in practical classes | **1** | **2** | **3** | **4** | **5** | **6** | **7** |
| **10.** With DSM it is easier to learn in practical classes | **1** | **2** | **3** | **4** | **5** | **6** | **7** |
| **11.** I find DSM useful for my study projects | **1** | **2** | **3** | **4** | **5** | **6** | **7** |
| **12.** DSM allows me to do a better job of my project | **1** | **2** | **3** | **4** | **5** | **6** | **7** |
| **13.** I find DSM easy to use in practical classes | **1** | **2** | **3** | **4** | **5** | **6** | **7** |
| **14.** With DSM it is easier to access teaching materials | **1** | **2** | **3** | **4** | **5** | **6** | **7** |
| **15.** I use DSM in practical classes because the other students use them | **1** | **2** | **3** | **4** | **5** | **6** | **7** |
| **16.** Using DSM makes me more efficient | **1** | **2** | **3** | **4** | **5** | **6** | **7** |
| **17.** DSM in practical classes is useful for my training | **1** | **2** | **3** | **4** | **5** | **6** | **7** |
| **18**. With DSM I have a better understanding of my study project | **1** | **2** | **3** | **4** | **5** | **6** | **7** |

**Additional file 2: Situational motivation scale (SIMS)**

**French version used**

Lisez chaque affirmation avec attention. Puis, en utilisant l’échelle suivante, entourez le nombre qui décrit le mieux à quel point vous êtes en accord avec chaque affirmation.

**Utilisez l’échelle ci- dessous :**

| **Pas du tout d’accord**  **1** | **Très peu d’accord**  **2** | **Un peu d’accord**  **3** | **Moyennement d’accord**  **4** | **Assez d’accord**  **5** | **Fortement d’accord**  **6** | **Tout à fait d’accord**  **7** |
| --- | --- | --- | --- | --- | --- | --- |

**Etes-vous :**

- Une femme
- Un homme

**Avez-vous réalisé :**

- Le TP traditionnel
- Le TP numérique

| ***Pourquoi participez-vous à cette séance ?*** | | | | | | | |
| --- | --- | --- | --- | --- | --- | --- | --- |
| **1.** Parce que cette activité est vraiment plaisante. | **1** | **2** | **3** | **4** | **5** | **6** | **7** |
| **2.** Parce que j’ai choisi d’y participer pour mon bien. | **1** | **2** | **3** | **4** | **5** | **6** | **7** |
| **3.** Parce que je suis supposé(e) la faire. | **1** | **2** | **3** | **4** | **5** | **6** | **7** |
| **4.** Je ne sais pas ; je ne vois pas ce que cela me procure. | **1** | **2** | **3** | **4** | **5** | **6** | **7** |
| **5.** Parce que je me sens bien en faisant cette activité. | **1** | **2** | **3** | **4** | **5** | **6** | **7** |
| **6.** Parce que je crois que cette activité est importante pour moi. | **1** | **2** | **3** | **4** | **5** | **6** | **7** |
| **7.** Parce que c’est quelque chose que je dois faire. | **1** | **2** | **3** | **4** | **5** | **6** | **7** |
| **8.** Je fais cette activité, mais je ne suis pas sûr(e) que cela en vaut la peine. | **1** | **2** | **3** | **4** | **5** | **6** | **7** |
| **9.** Parce que je trouve cette activité intéressante. | **1** | **2** | **3** | **4** | **5** | **6** | **7** |
| **10.** Parce que je sens que je veux faire cette activité. | **1** | **2** | **3** | **4** | **5** | **6** | **7** |
| **11.** Parce que c’est quelque chose que je dois faire. | **1** | **2** | **3** | **4** | **5** | **6** | **7** |
| **12.** Je fais cette activité, mais en me demandant si je dois la faire. | **1** | **2** | **3** | **4** | **5** | **6** | **7** |
| **13.** Parce que je trouve cette activité agréable. | **1** | **2** | **3** | **4** | **5** | **6** | **7** |
| **14.** Parce que je trouve que participer à cette activité est bon pour moi. | **1** | **2** | **3** | **4** | **5** | **6** | **7** |
| **15.** Parce que je sens que je n’ai pas d’autres choix que de la faire. | **1** | **2** | **3** | **4** | **5** | **6** | **7** |
| **16.** Il y a peut-être de bonnes raisons pour faire cette activité mais, personnellement, je n’en vois pas. | **1** | **2** | **3** | **4** | **5** | **6** | **7** |

**English translation of the French version**

Read each statement carefully. Then, using the following scale, circle the number, which best describes the extent that you agree with each statement.

**Use the scale below:**

| **Completely disagree** | **Strongly disagree** | **Somewhat disagree** | **Neither agree nor disagree** | **Somewhat agree** | **Strongly agree** | **Completely**  **agree** |
| --- | --- | --- | --- | --- | --- | --- |
| **1** | **2** | **3** | **4** | **5** | **6** | **7** |

**Are you:**

- Female
- Male

**Did you take part in:**

- Traditional practical class
- Digital practical class

| ***Why did you participate in the chosen class?*** | | | | | | | |
| --- | --- | --- | --- | --- | --- | --- | --- |
| **1.** Because the activity was really pleasant | **1** | **2** | **3** | **4** | **5** | **6** | **7** |
| **2.** I chose to participate because I thought it would be good for me | **1** | **2** | **3** | **4** | **5** | **6** | **7** |
| **3.** Because I was supposed to do it | **1** | **2** | **3** | **4** | **5** | **6** | **7** |
| **4.** I do not know, I don’t see what I will gain from it | **1** | **2** | **3** | **4** | **5** | **6** | **7** |
| **5.** Because I feel good doing this activity | **1** | **2** | **3** | **4** | **5** | **6** | **7** |
| **6.** Because I think this activity is important for me | **1** | **2** | **3** | **4** | **5** | **6** | **7** |
| **7.** Because it is something that I was obliged to do | **1** | **2** | **3** | **4** | **5** | **6** | **7** |
| **8.** I did this activity but I am not sure that it was worthwhile | **1** | **2** | **3** | **4** | **5** | **6** | **7** |
| **9.** Because I find this activity interesting | **1** | **2** | **3** | **4** | **5** | **6** | **7** |
| **10.** Because I feel that I want to take part in this activity | **1** | **2** | **3** | **4** | **5** | **6** | **7** |
| **11.** Because it is something that I have to do | **1** | **2** | **3** | **4** | **5** | **6** | **7** |
| **12.** I have to do this activity but I wonder why I have to do it | **1** | **2** | **3** | **4** | **5** | **6** | **7** |
| **13.** Because I find this activity enjoyable | **1** | **2** | **3** | **4** | **5** | **6** | **7** |
| **14.** Because I find taking part in this activity is good for me | **1** | **2** | **3** | **4** | **5** | **6** | **7** |
| **15.** Because I feel like I do not have any choice but to do it | **1** | **2** | **3** | **4** | **5** | **6** | **7** |
| **16.** There may be good reasons to do this activity but personally I don’t see them | **1** | **2** | **3** | **4** | **5** | **6** | **7** |

**Additional file 3: Evaluation of motivation after the activity based on the theory of self-determination of Deci and Ryan (2002) and adapted for animal experimentation in pharmacology**

**French version used**

Lisez chaque affirmation avec attention. Puis, en utilisant l’échelle suivante, entourez le nombre qui décrit le mieux à quel point vous êtes en accord avec chaque affirmation.

**Utilisez l’échelle ci- dessous :**

| **Pas du tout d’accord**  **1** | **Très peu d’accord**  **2** | **Un peu d’accord**  **3** | **Moyennement d’accord**  **4** | **Assez d’accord**  **5** | **Fortement d’accord**  **6** | **Tout à fait d’accord**  **7** |
| --- | --- | --- | --- | --- | --- | --- |

**Etes-vous :**

- Une femme
- Un homme

**Avez-vous réalisé :**

- Le TP traditionnel
- Le TP numérique

***Qu’en pensez-vous ?***

| **1.** J’apprends de nouvelles choses en réalisant des expériences sur les animaux. | **1** | **2** | **3** | **4** | **5** | **6** | **7** |
| --- | --- | --- | --- | --- | --- | --- | --- |
| **2.** C’est pour moi une satisfaction de réaliser un TP sous format numérique. | **1** | **2** | **3** | **4** | **5** | **6** | **7** |
| **3.** C’est pour moi un défi de réaliser une expérimentation sur un animal. | **1** | **2** | **3** | **4** | **5** | **6** | **7** |
| **4.** C’est plus stimulant d’effectuer un TP numérique. | **1** | **2** | **3** | **4** | **5** | **6** | **7** |
| **5.** Je réalise des TP sur des animaux car cela correspond à mes valeurs. | **1** | **2** | **3** | **4** | **5** | **6** | **7** |
| **6.** J’estime important de ne plus travailler sur des animaux lors des TP. | **1** | **2** | **3** | **4** | **5** | **6** | **7** |
| **7.** J’apprécie la façon dont le numérique amène à des changements dans ma vie professionnelle | **1** | **2** | **3** | **4** | **5** | **6** | **7** |
| **8.** J’ai conscience du mal que je fais aux animaux dans les TP traditionnels. | **1** | **2** | **3** | **4** | **5** | **6** | **7** |
| **9.** Il est important pour moi de réaliser le TP avec les méthodes traditionnelles. | **1** | **2** | **3** | **4** | **5** | **6** | **7** |
| **10.** Il est important pour moi de ne plus toucher aux animaux lors d’un TP. | **1** | **2** | **3** | **4** | **5** | **6** | **7** |
| **11.** En tant qu’étudiant(e), je dois participer à des expériences sur des animaux. | **1** | **2** | **3** | **4** | **5** | **6** | **7** |
| **12.** Je préfère réaliser les TP numériques car selon mon entourage, c’est mieux pour les animaux. | **1** | **2** | **3** | **4** | **5** | **6** | **7** |
| **13.** Je suis contraint(e) de réaliser le TP sur des animaux alors que je préférerais m’abstenir. | **1** | **2** | **3** | **4** | **5** | **6** | **7** |
| **14.** Je manipule des animaux pour montrer aux autres que je suis autant capable qu’eux. | **1** | **2** | **3** | **4** | **5** | **6** | **7** |
| **15.** Je réalise des expériences sur des animaux pour me prouver que je suis capable. | **1** | **2** | **3** | **4** | **5** | **6** | **7** |
| **16.** Je fais ce TP sur des animaux parce que je n’ai pas le choix. | **1** | **2** | **3** | **4** | **5** | **6** | **7** |
| **17.** Je suis obligé(e) de réaliser des TP numériques alors que j’aurai préféré employer des méthodes traditionnelles. | **1** | **2** | **3** | **4** | **5** | **6** | **7** |
| **18.** Je dois réaliser des expérimentations sur des animaux afin de donner une bonne impression de moi. | **1** | **2** | **3** | **4** | **5** | **6** | **7** |
| **19.** Je ne vois pas pourquoi il faut réaliser des TP sans les animaux. | **1** | **2** | **3** | **4** | **5** | **6** | **7** |
| **20.** Je ne vois pas ce que le numérique apporte dans les TP. | **1** | **2** | **3** | **4** | **5** | **6** | **7** |
| **21.** C’est démotivant de faire des expérimentations sur des animaux. | **1** | **2** | **3** | **4** | **5** | **6** | **7** |

**English translation of the French version**

Read each statement carefully. Then, using the following scale, circle the number, which best describes the extent that you agree with each statement.

**Use the scale below:**

| **Completely disagree** | **Strongly disagree** | **Somewhat disagree** | **Neither agree nor disagree** | **Somewhat agree** | **Strongly agree** | **Completely**  **agree** |
| --- | --- | --- | --- | --- | --- | --- |
| **1** | **2** | **3** | **4** | **5** | **6** | **7** |

**Are you:**

- Female
- Male

**Did you take part in:**

- Traditional practical class
- Digital practical class

***What did you think?***

| **1.** I learn new things by doing practical classes with animals | **1** | **2** | **3** | **4** | **5** | **6** | **7** |
| --- | --- | --- | --- | --- | --- | --- | --- |
| **2.** It was satisfying for me to do practical class in a digital format | **1** | **2** | **3** | **4** | **5** | **6** | **7** |
| **3.** It’s a challenge for me to carry out an experiment using an animal | **1** | **2** | **3** | **4** | **5** | **6** | **7** |
| **4.** It’s more exciting to do a digital practical class | **1** | **2** | **3** | **4** | **5** | **6** | **7** |
| **5.** I do practical classes with animals as that matches my values | **1** | **2** | **3** | **4** | **5** | **6** | **7** |
| **6.** I think it is important to no longer work on animals in practical classes | **1** | **2** | **3** | **4** | **5** | **6** | **7** |
| **7.** I like the way in which digital resources have changed my professional life | **1** | **2** | **3** | **4** | **5** | **6** | **7** |
| **8.** I am conscious of the harm that I am doing to animals in traditional practical classes | **1** | **2** | **3** | **4** | **5** | **6** | **7** |
| **9.** It is important for me to participate in practical classes using traditional methods | **1** | **2** | **3** | **4** | **5** | **6** | **7** |
| **10.** It is important for me to no longer handle animals during practical classes | **1** | **2** | **3** | **4** | **5** | **6** | **7** |
| **11.** As a student, I have to participate in animal experiments | **1** | **2** | **3** | **4** | **5** | **6** | **7** |
| **12.** I prefer to do digital practical classes as, according to my friends, it is better for animals | **1** | **2** | **3** | **4** | **5** | **6** | **7** |
| **13.** I am required to do practical classes using animals but I would prefer not to | **1** | **2** | **3** | **4** | **5** | **6** | **7** |
| **14.** I handle the animals to show others that I am just as capable as they are | **1** | **2** | **3** | **4** | **5** | **6** | **7** |
| **15.** I perform experiments on animals to show myself that I am capable | **1** | **2** | **3** | **4** | **5** | **6** | **7** |
| **16**. I do practical classes with animals because I have no choice | **1** | **2** | **3** | **4** | **5** | **6** | **7** |
| **17.** I am obliged to do digital practical classes but I would prefer to use traditional methods | **1** | **2** | **3** | **4** | **5** | **6** | **7** |
| **18.** I have to do animal experiments to give a good impression | **1** | **2** | **3** | **4** | **5** | **6** | **7** |
| **19.** I do not see why it is necessary to do practical classes without animals | **1** | **2** | **3** | **4** | **5** | **6** | **7** |
| **20.** I do not see what the use of digital tools brings to practical classes | **1** | **2** | **3** | **4** | **5** | **6** | **7** |
| **21.** It’s demotivating to do experiments on animals | **1** | **2** | **3** | **4** | **5** | **6** | **7** |

**Additional file 4: General knowledge evaluation and final examination**

**(Total exam duration: 30 minutes)**

**Part 1: General knowledge evaluation**

**Question 1:** Regarding the kidney, which of the following statements are correct?

1. The adrenal cortex is located above the kidney
2. The functional unit of the kidney is the nephron
3. The nephron consists of a glomerulus and tubules
4. The glomerulus filters the blood
5. The ion composition of the ultrafiltrate is modified in the tubules

Answers: A, B, C, D, E

**Question 2:** Regarding diuresis, which of the following statements are correct?

1. Physiological diuresis in human is between 800 and 1,500 ml per 24 h
2. It can vary according to the quantity of water absorbed (drinks, foods), the level of perspiration (surrounding temperature, physical efforts)
3. Diuretics decrease diuresis
4. Diuretics are natriuretic
5. Diuresis is measured in the laboratory by measuring the total volume of urine produced over a 24-hour period. The urine is collected at each micturition

Answers: A, B, D, E

**Question 3:** Regarding diuretics, which of the following statements are correct?

1. Diuretics are indicated in hypertension
2. Diuretics increase urinary sodium excretion
3. Diuretics are classified according to their mode of action and their site of action
4. Diuretics are indicated in edema of cardiac origin
5. Diuretics are indicated in angina

Answers: A, B, C, D

**Question 4:** Regarding diuretics, which of the following statements are correct?

1. Loop diuretics have moderate efficacy
2. Thiazide diuretics are highly effective
3. Spironolactone is a potassium-sparing diuretic
4. Amiloride is a diuretic of the “pseudoantialdosterone” class
5. Furosemide is an osmotic diuretic

Answers: C, D

**Question 5:** Regarding loop diuretics, which of the following statements are correct?

1. They act on ascending branch of the loop of Henle
2. They inhibit by competition with the chlorine site, the NKCC co-transporter located on the apical membrane of the renal cell
3. They induce hyponatremia and hyperkalemia
4. NSAIDs increase their diuretic effect
5. They increase the calcemia and the excretion of magnesium ions

Answers: A, B, E

**Part 2: Final examination**

**Question 1: Dose calculation exercise**

You need to prepare the furosemide (20 mg/kg) gavage solution for the “furosemide” group of 10 rats. You have ampoules of furosemide at 20 mg/2 ml. The individual weights of the rats are the followings: 230, 202, 220, 247, 225, 250, 215, 210, 205 and 234.

A volume of 0.5 ml per 100 g of rat is required for the gavage solution.

1. How many ampoules are you going to use?
2. What volume of solvent is needed to obtain the concentration and volume of the gavage solution to be prepared for this batch?

Answer:

Furosemide 20 mg/1000 g

Total weight of rats: 2238 g (we will prepare sufficient volume for 2500 g of rats)

1. If we need 20 mg/ 1000g, we will need a total of 50 mg for 2500 g or **5 ml of drug**. Therefore, **we will use equivalent volume of 2.5 ampoules.**
2. If we need 0.5 ml/100 g, **we will need a total of 12.5 ml for 2500 g**.

So 50 mg for a total volume of 12.5 ml. We will use a mix of **5 ml of furosemide dissolved in 7.5 ml of saline** to prepare the solution

**Question 2: Analyze the graph below and answer the following questions**

1. Propose a title with detailed legend to this graph

Additional information: Fur (furosemide); HG (high-grown black tea infusion of Sri Lanka); urine output, ml/100 g BW (body weight) of a rat; 10 rats/groups; * p<0.05 versus Control

Answer: “Diuretic effect of High-Grown black tea of Sri Lanka every hour for 6 hours after administration of 3 doses (300; 600 and 2400 mg/kg) in rats in comparison to Control and furosemide treated groups”. Fur (furosemide), N = 10/ group, *p<0.05 versus control.

1. Describe this graph

Answer: “The 3 doses of High-Grown black tea of Sri Lanka have a greater diuretic effect than the control group but less than furosemide at 13 mg/kg”.

1. What was the scientific question or hypothesis that drives this experiment?

Answer: “Does High-Grown black tea of Sri Lanka have a diuretic effect?”

1. What groups of animals were needed to obtain these results?

Answer: 5 groups:

- Control (vehicle)
- High-Grown black tea of Sri Lanka (300 mg/kg)
- High-Grown black tea of Sri Lanka (600 mg/kg)
- High-Grown black tea of Sri Lanka (2400 mg/kg)
- Furosemide (13 mg/kg)

1. How did they measure urine output at for example 1, 2 and 3 hours?

Answer:

For 1h: (urinary volume excreted at 1h/100g)/1h

For 2h: (urinary volume excreted at 2h/100g - urinary volume excreted at 1h/100g)/1h

For 3h: (urinary volume excreted at 3h/100g - urinary volume excreted at 2h/100g)/1h

1. Name 4 materials or tools needed for this experiment.

Answer: “Metabolic cages, rats, gavage material, gavage solution”
